# Noninvasive Evaluation of Bladder Wall Mechanical Properties as a Function of Filling Volume: Potential Application in Bladder Compliance Assessment

**DOI:** 10.1371/journal.pone.0157818

**Published:** 2016-06-24

**Authors:** Ivan Nenadic, Lance Mynderse, Douglas Husmann, Mohammad Mehrmohammadi, Mahdi Bayat, Aparna Singh, Max Denis, Matthew Urban, Azra Alizad, Mostafa Fatemi

**Affiliations:** 1 Department of Physiology and Biomedical Engineering, Mayo Clinic College of Medicine, Rochester, Minnesota, United States of America; 2 Department of Urology, Mayo Clinic College of Medicine, Rochester, Minnesota, United States of America; 3 Department of Radiology, Mayo Clinic College of Medicine, Rochester, Minnesota, United States of America; Politecnico di Milano, ITALY

## Abstract

**Purpose:**

We propose a novel method to monitor bladder wall mechanical properties as a function of filling volume, with the potential application to bladder compliance assessment. The proposed ultrasound bladder vibrometry (UBV) method uses ultrasound to excite and track Lamb waves on the bladder wall from which its mechanical properties are derived by fitting measurements to an analytical model. Of particular interest is the shear modulus of bladder wall at different volumes, which we hypothesize, is similar to measuring the compliance characteristics of the bladder.

**Materials and Methods:**

Three experimental models were used: 1) an ex vivo porcine model where normal and aberrant (stiffened by formalin) bladders underwent evaluation by UBV; 2) an in vivo study to evaluate the performance of UBV on patients with clinically documented compliant and noncompliant bladders undergoing UDS; and 3) a noninvasive UBV protocol to assess bladder compliance using oral hydration and fractionated voiding on three healthy volunteers.

**Results:**

The ex vivo studies showed a high correlation between the UBV parameters and direct pressure measurement (*R*^2^ = 0.84–0.99). A similar correlation was observed for 2 patients with compliant and noncompliant bladders (*R*^2^ = 0.89–0.99) undergoing UDS detrusor pressure-volume measurements. The results of UBV on healthy volunteers, performed without catheterization, were comparable to a compliant bladder patient.

**Conclusion:**

The utility of UBV as a method to monitor changes in bladder wall mechanical properties is validated by the high correlation with pressure measurements in ex vivo and in vivo patient studies. High correlation UBV and UDS in vivo studies demonstrated the potential of UBV as a bladder compliance assessment tool. Results of studies on healthy volunteers with normal bladders demonstrated that UBV could be performed noninvasively. Further studies on a larger cohort are needed to fully validate the use of UBV as a clinical tool for bladder compliance assessment.

## Introduction

The normal urinary bladder is a highly compliant elastic organ capable of storing urine at low pressures [1]. The ability of the bladder to stretch in volume without a significant change in pressure is called *detrusor compliance*. Detrusor compliance is based on the physiological balance of the bladders muscular layer and fibrous connective tissue. In the presence of neurologic injuries, chronic infection, or urinary outlet obstruction there is an increase in the percent of connective tissue in the bladder interstitium compared to smooth muscle. This increase in connective tissue can result in the bladder becoming more rigid and reducing its ability to store urine with low pressure. This pathologic might lead to both urinary incontinence and upper tract deterioration [2,3,4]. The unique and variable time-course of many of these conditions require repeated assessments of the bladder compliance to properly manage these disorders and limit the negative effects on renal function over time.

Currently, urodynamic studies (UDS) is the clinical gold standard for the assessment of bladder compliance. A typical UDS procedure lasts approximately 45 minutes, requires catheter placement in the bladder and the vagina or rectum, and filling the bladder at a defined rate via a fluid pump [5]. By determining the 2 pressure measurements, i.e., the bladder and abdominal pressure, detrusor pressure and subsequently detrusor compliance can be determined. The relationship between the change in bladder volume and detrusor pressure describes detrusor compliance. In the case of a compliant bladder, the bladder wall expands to accommodate the filling volume resulting in a low detrusor pressure. Non-compliant bladders will exhibit considerable increases in detrusor pressure during bladder filling [4]. There is a rare occurrence of complications from urinary catheterization associated with UDS [6,7]. Having a reasonably simple, non-operator dependent and potentially noninvasive option to evaluate and track bladder compliance would be an invaluable and cost effective tool.

Acoustic radiation force (ARF) and fast ultrasound imaging have enabled a variety of techniques for noninvasive tissue characterization based on shear wave speed [8,9,10,11,12,13] and shear wave dispersion analysis [14]. The application of such techniques in vivo has shown promising results in the differentiation of lesions in a number of organs, especially in breast and thyroid [8,10]. The requirements of such techniques is the availability of an acoustic window for coupling the radiation force to the medium, as well as simultaneous tracking of the induced waves using ultrasound. Ultrasound bladder vibrometry (UBV) uses the same concepts to excite a Lamb wave in the bladder wall using an ARF beam [15,16] from which mechanical properties are derived by fitting measurements to an analytical model.

The aim of this paper is to examine the feasibility of UBV as a technique for monitoring changes to the bladder wall mechanical properties. To this end, the following experiments were conducted: 1) an ex vivo study is performed correlating UBV parameters to the direct bladder pressure measurement as a function of filling volume in a physiologically normal and aberrant animal model; 2) an initial proof of concept study is performed to correlate UBV parameters with UDS pressure measurements to determine its ability to monitor 2 different in vivo bladder compliance conditions (compliant and noncompliant); and 3) UBV measurements are performed on healthy human volunteers using oral fluid hydration and fractionated voiding to demonstrate that UBV can be performed noninvasively without requiring a catheter to fill the bladder.

## Materials and Methods

### Principles of Ultrasound Bladder Vibrometry

**Fig 1:**
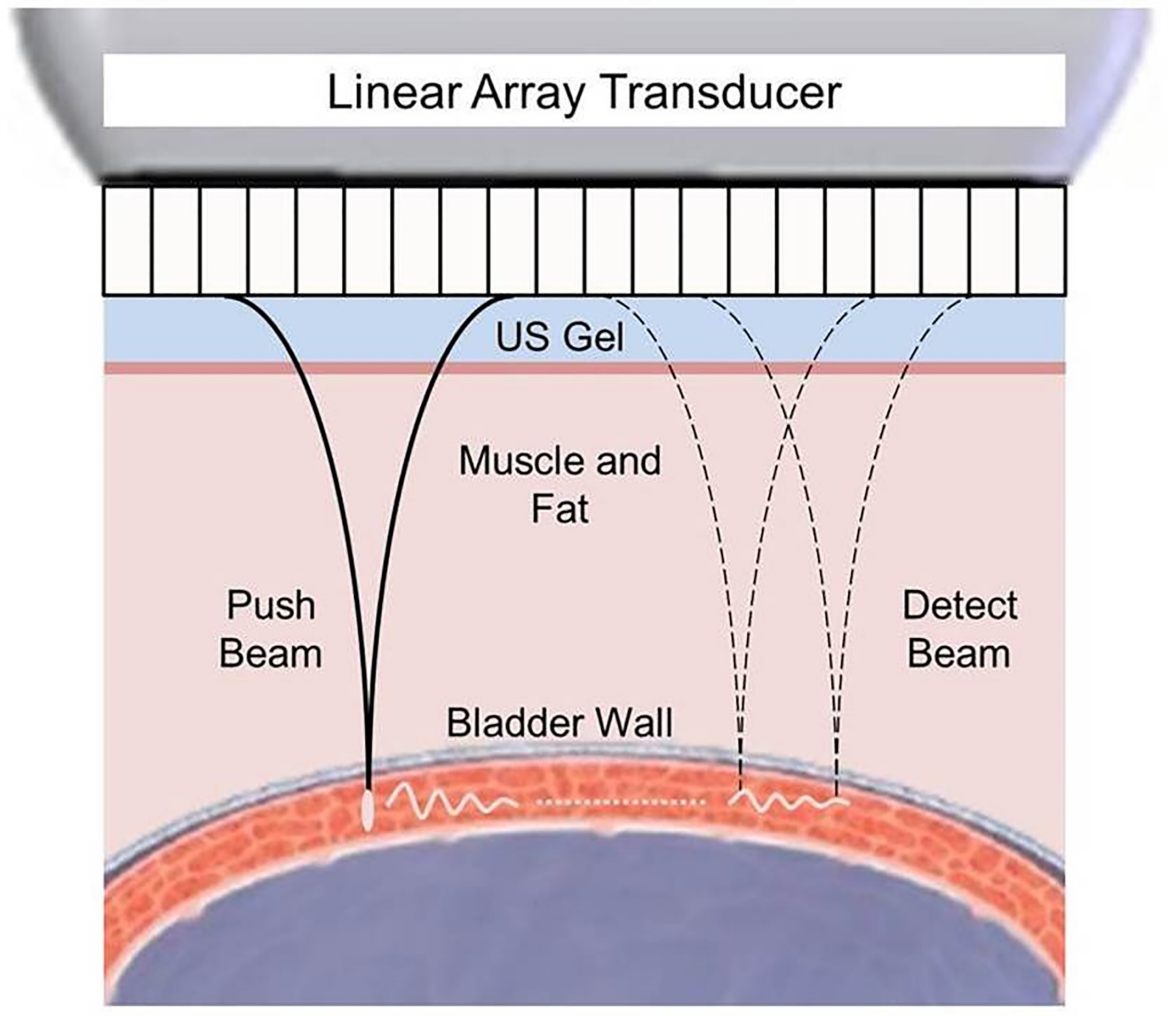
An ultrasound array transducer is utilized to generate an ARF beam (push beam) on the bladder wall, generating an anti-symmetric Lamb wave displacement field. The displacement field is tracked by a pulse-echo detection beam (detect beam). In order to identify the bladder wall, a line is drawn along the bladder wall on the B-mode image. The displacement field is measured along this line. To obtain appropriate coupling between the transducer and skin surface above the bladder, standard ultrasound coupling gel (US gel) is utilized.

The concept of UBV is to utilize an ultrasound array transducer to generate an ARF beam on the bladder wall (push beam) that excites anti-symmetric Lamb waves in Fig 1. Pulse-echo ultrasound (detect beam) is then used to track the motion of the Lamb wave in the bladder wall. We modeled the bladder wall as an incompressible, homogenous, isotropic solid plate submerged in an incompressible nonviscous fluid [16]. For an in vivo application, the urine below the bladder wall would satisfy the incompressible nonviscous fluid assumption. Meanwhile, the fascial-defined space above the bladder (mainly composing of mostly fluid and fat), has demonstrated minimal effects on the appropriateness of the Lamb wave model [15]. The anti-symmetric Lamb wave dispersion equation for such a geometry is [15,16]:

$$4k_{L}^{3}\beta \;\text{tanh}\left(\beta h\right)=\;k_{s}^{4}+\left(k_{s}^{2}-k_{L}^{2}\right)\;\text{tanh}\left(k_{L}h\right)$$ (1)

where $\beta =\;\sqrt{k_{L}^{2}-k_{s}^{2}}$, *k*_*L*_ *= ω/c* is the Lamb wave number, *ω* is the angular frequency, *c* is the frequency dependent Lamb wave phase velocity, $k_{s}=\omega \sqrt{\rho /\mu }$ is the shear wave number, *ρ* is the tissue density, *μ = c*^*2*^ */ ρ* is the bladder wall shear modulus, and *h* is the half-thickness of the wall. While the surface of the bladder is curved, the pulse-echo technique (detect beam) measures the axial motion of the propagating shear wave, i.e., the component of tissue motion parallel to the excitation. To correct for the curvature of the bladder wall, the displacement vector *x* was constructed by recognizing the arc length *x = rθ*, where *r* is the radius of curvature and *θ* is the angle.

**Fig 2:**
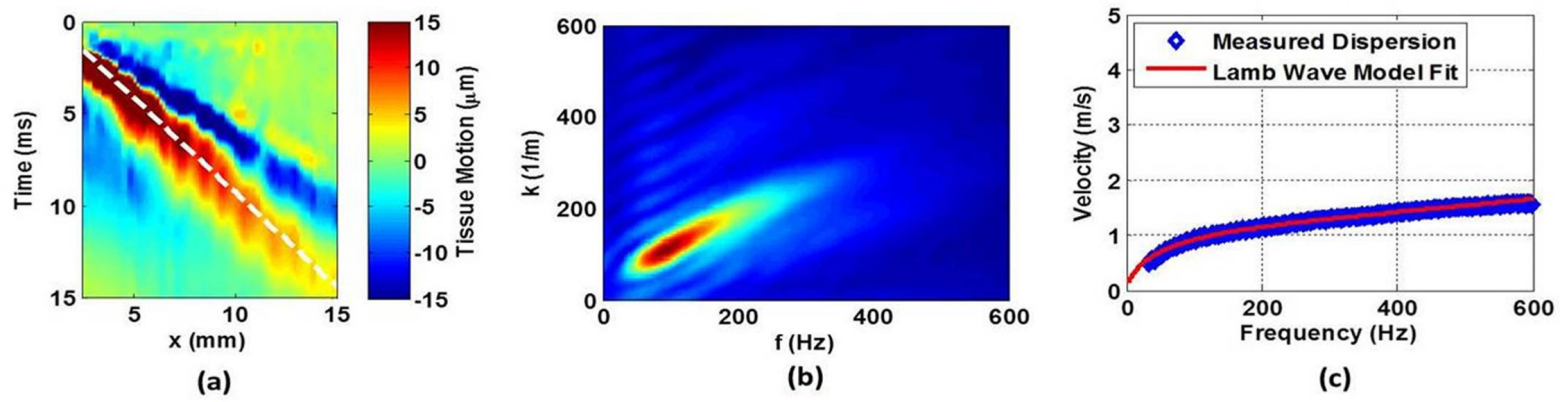
(a) The bladder wall displacement as a function of time and distance. Group velocity is calculated from the slope of the dashed line. (b) A 2D FFT of the bladder wall displacement results in the k-space representation of the measured UBV data. (c) Lamb wave dispersion curve of the measured data and the analytical fit.

The procedures for estimating bladder wall UBV parameters of group velocity and shear modulus are outlined in Fig 2. In order to analyze motion along the bladder wall, a line is drawn along the bladder wall from the B-mode image. Fig 2A shows the bladder displacement as a function of time (*t*) and distance (*x*) calculated using a cross-spectral analysis [17]. The group velocity (*c*_*g*_) is obtained by tracking the displacement peaks and calculating the slope (*c*_*g*_ *= dx/dt)* of the line. A two-dimensional Fast Fourier Transform (FFT) of the displacement data yields the k-space map shown in Fig 2B, whose coordinates are frequency (*f*) and wave number (*k*). In Fig 2C, a peak-searching approach is employed to obtain the experimental Lamb wave phase velocity (*c* = *k*/*f*) at each frequency, i.e. the Lamb wave dispersion curve. The Lamb wave dispersion equation was fit to the dispersion data to estimate the shear modulus of elasticity (*μ)*. The thickness of the bladder wall was estimated from the B-mode ultrasound image.

### UBV and Direct Pressure Measurements for Formalin Treated and Untreated Ex Vivo Bladders

For the ex vivo experiments, three porcine bladders were obtained from the Department of Surgery, Mayo Clinic Rochester, Minnesota. The pigs were sacrificed by the Mayo Clinic Department of Surgery under an approved surgical training protocol #A7013 assigned from the Mayo Clinic Institutional Animal Care and Use Committee using either exsanguination or Fatal Plus.

Following an excision, the ex vivo porcine bladders were emptied and washed with saline through the urethra. To prevent leaks while filling the porcine bladder, the ureters were tied. Also, cyanoacrylate glue was used to cover the inner layer of the urethra. The bladders were filled through the rubber tubing attached to the urethra while ensuring that the water could not leak through the interface. The experiments were conducted in 2 parts. The first part of the experiment was performed on the bladder in its initial state. For the second part of the experiment, the porcine bladder was treated with formalin to artificially induce tissue stiffening. Changes in the bladder group velocity and shear modulus versus pressure and volume were measured before and after formalin treatment. It is noted that tissue stiffening due to formalin does not necessarily emulate tissue stiffening due to pathology. Formalin was mainly used to stiffen the tissue for the purpose of demonstrating the ability to detect changes in tissue stiffness using UBV.

For the UBV experiments, we employed a fully programmable ultrasound imaging system (Verasonics, Kirkland, WA, USA) and a 128-element linear array L7-4 transducer with the center frequency of 5 MHz (Philips Healthcare, Andover, MA). This system generated the ARF beam in the form of a 400 μs toneburst. The ARF beam was focused on the bladder wall to induce a Lamb wave in the bladder wall. The toneburst length (400 μs) was chosen to ensure sufficient tissue motion. The same transducer was used in pulse echo mode to track the wave motion of the bladder wall. Plane wave imaging detection pulses were transmitted at a pulse repetition frequency of 4 kHz. The pulse repetition frequency of the detect pulses was chosen to ensure enough samples to resolve wave dispersion up to 500 Hz.

For the first part of the experiment, water was used to fill the bladder with approximately 200 mL of initial volume. The volume of water inside the bladder was recorded and then the bladder was placed inside of a water tank for UBV measurements. To change the pressure of the bladder a vertical water-column was used, and to change the volume a syringe system was employed. To measure the pressure inside the bladder a pressure gauge (Omegadyne, Inc., Sunbury, OH, USA) was used. Bladder volume varied from 200 mL to 280 mL in increments of 10 mL and the pressure at each volume was recorded. Thereafter, five UBV measurements were performed at each incremental volume.

For the second experiment, the bladder was emptied and injected with 10% formalin solution (Fisher Scientific Co, LLC, Hanover Park, IL). The formalin-injected bladder was then submerged into the same formalin solution for 120 minutes. After the formalin treatment, the bladder was rinsed with saline, filled with water, and placed back into the water tank. Thereafter, UBV measurements were conducted similar to the first experiment.

### In Vivo UDS and UBV Patient Measurements

Concurrent UDS and UBV measurements were conducted on *n = 2* patients. Inclusion criteria required that patient participants must be 21 years of age or older, and scheduled to undergo UDS for their clinical care. Participants must also have at least 1 of the following conditions: neurogenic bladder, stress incontinence or benign prostatic hyperplasia. The exclusion criteria were: obese patients (body mass index>35), known neurologic disease other than that mentioned, prolonged catheter drainage, previous pelvic radiation therapy, previous radical pelvic surgery (such as for colon cancer), prior bladder surgery (including prostate resection) and pregnant or breast-feeding women. Both patients had spinal cord injuries; however, one was known to have normal bladder compliance and the other had been clinically documented as noncompliant. The studies were performed in accordance to a protocol approved by the Mayo Clinic Institutional Review Board. Informed written consent was obtained from all participants.

**Fig 3:**
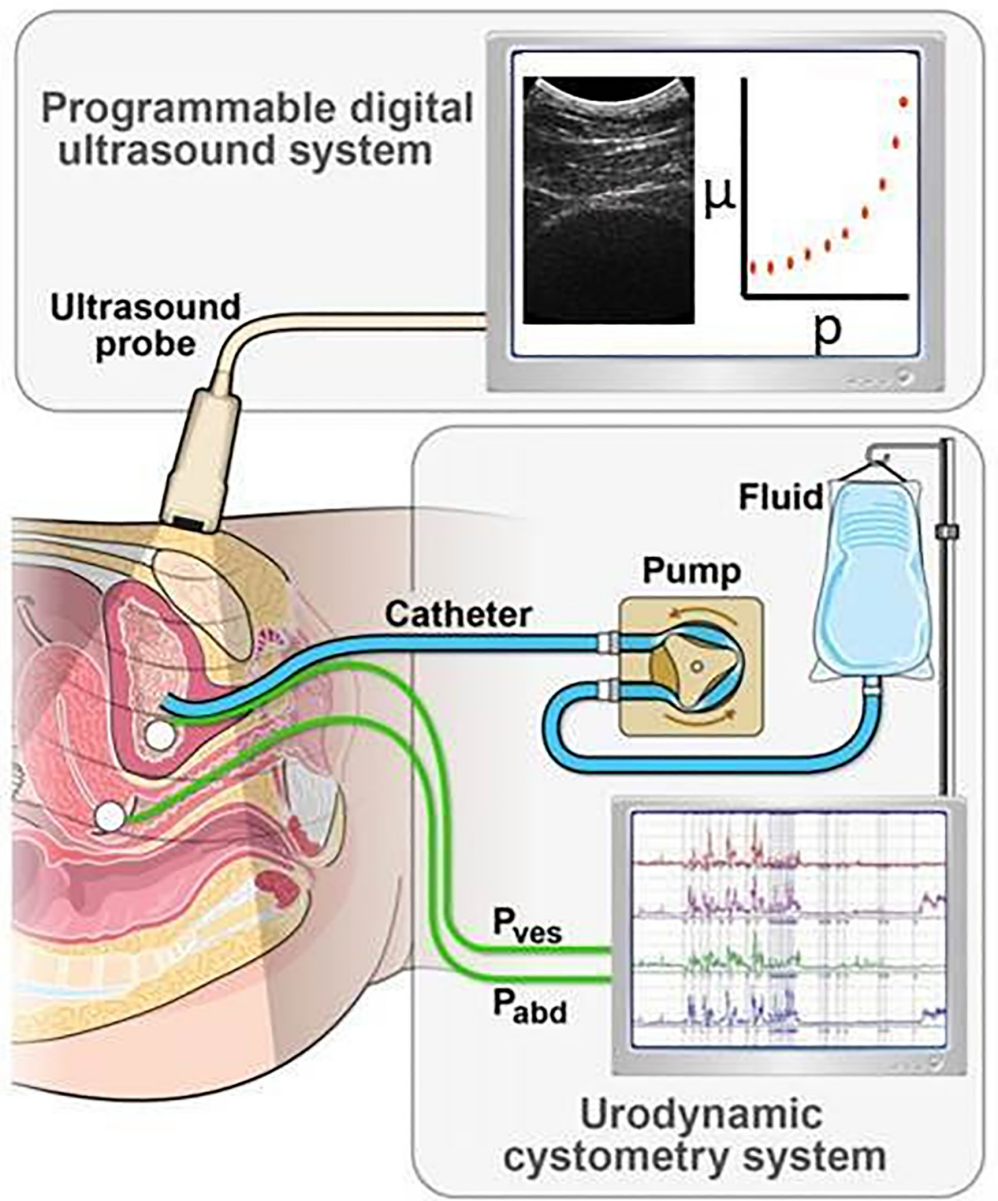
Diagram of the in vivo patient studies depicting the concurrent UDS cystometry and UBV measurements, where *P*_ves_ = vesical pressure, *P*_abd_ = abdominal pressure, and *P*_det_ = *P*_ves_−*P*_abd_ = detrusor pressure.

Fig 3 depicts the concurrent UBV and UDS measurement procedure in our 2 patient studies. In UDS, one pressure sensor was inserted into the patient’s bladder and another into the rectum (or vagina). The difference in the pressure between the two sensors was used to calculate the detrusor pressure across the bladder wall. Saline was infused into the bladder through the catheter in volume increments. At each volume increment, detrusor pressure was recorded along with UBV measurements. The bladder wall was identified manually on the B-mode and targeted for focusing the push beam using an ultrasound system (Verasonics, Redmond, WA) equipped with a curved linear array (C4-2, ATL/Philips, Bothell, WA) whose center frequency is 2.5 MHz. An ultrasound toneburst 600–900 μs in duration was used to create the ARF beam to excite mechanical waves in the bladder wall. Plane wave imaging with a three-angle coherent angular compounding was used to track the wave propagation in the bladder wall [18]. Plane waves were transmitted with a pulse repetition frequency of 2.5 kHz at a center frequency of 3.0 MHz. The duration of the toneburst was chosen to create enough tissue motion. The imaging frame rate provided sufficient samples for continuous tracking of the induced Lamb waves up to 500Hz. The UBV data acquisition was approximately 100 ms; thus multiple acquisitions/volume could be acquired in less than a minute. The processing results were highly reproducible as the bladder wall was identified by a sonographer with more than 20 years of experience. All imaging and processing parameters were fixed for different patients to avoid retrospective tuning and calibrations.

### Healthy Human Volunteer UBV Studies without Catherization

Three healthy human volunteers with no history of bladder problems were recruited. The inclusion criteria required that the participants must be at least 21 and less than 40 years old. Volunteers consumed 0.5–1.0 liters of fluids (water or ice tea) and were asked to stop consuming food and drinks and refrain from voiding 2 hours before UBV studies. Prior to the first UBV measurements, the bladder volume was measured using the BladderScan device (Verathon, Bothell, WA). The participants were asked to void approximately 100 mL of urine at a time and UBV measurements were made after each incremental voiding. This process was repeated until all urine was voided. The voided volume was noted throughout the procedure and the BladderScan measurements were made at the time of each UBV measurement.

## Results

### Ex Vivo UBV Parameters and Their Correlation to Direct Pressure Measurements

**Fig 4:**
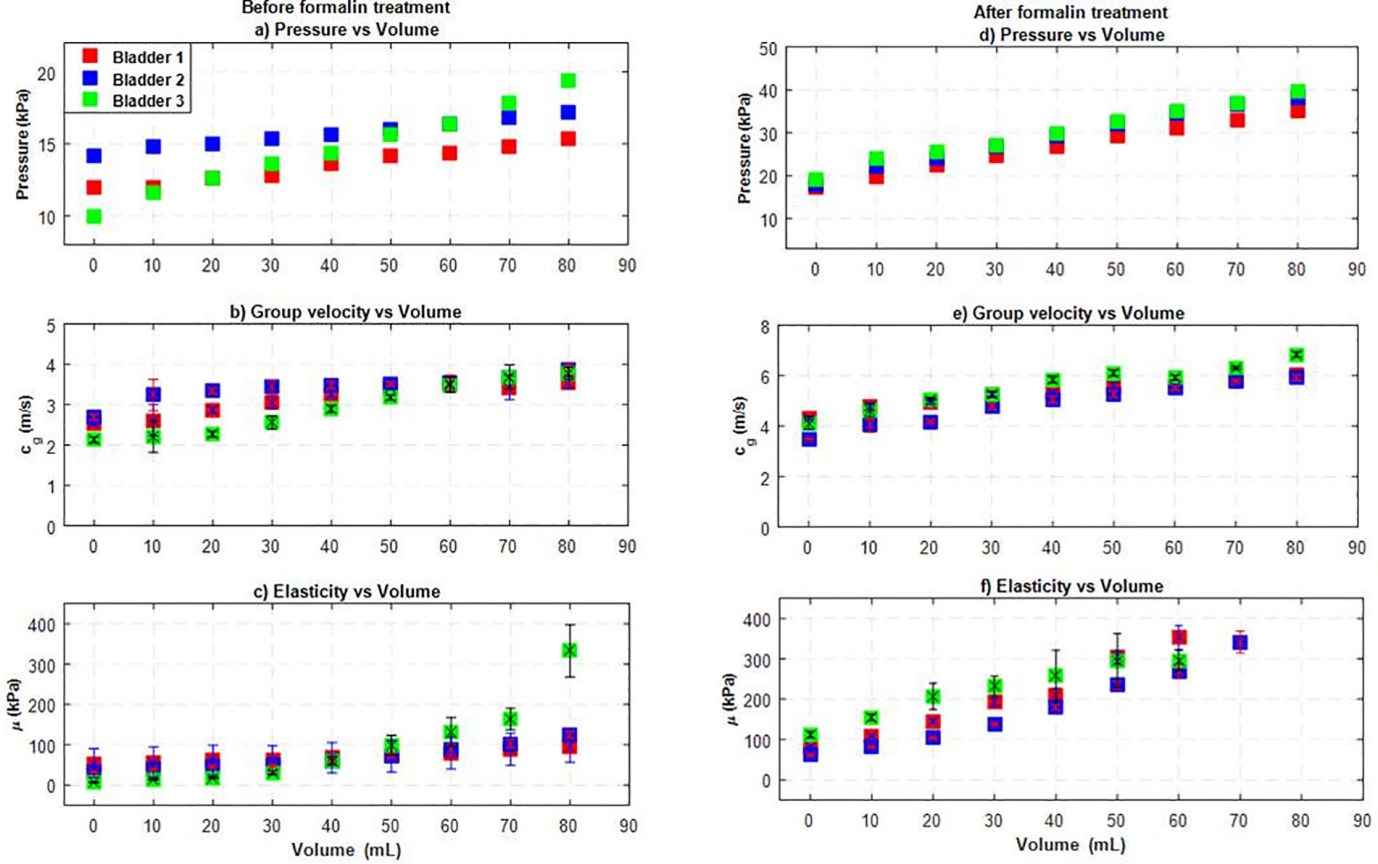
Summary of pressure, group velocity (*c*_*g*_), and shear modulus (*μ*) versus volume measurements (a-c) before and (d-f) after formalin treatment of three excised porcine bladders.

Following the procedures outlined for UBV, the porcine bladder pressure, group velocity and shear modulus were obtained at incremental filling volumes before and after formalin treatment. Fig 4A and 4D shows the pressure inside the bladder before and after the formalin treatment. The formalin treated bladder pressure has a higher slope than the untreated bladder, suggesting that formalin stiffened the tissue. Fig 4B and 4E show the group velocity of the waves on the bladder wall versus volume before and after the formalin treatment. The group velocity following the formalin treatment is higher and seems to increase as a function of volume. Fig 4C and 4F show the shear modulus of the bladder wall versus volume before and after the formalin treatment. The shear modulus increases more rapidly as a function of increasing filling volume following the formalin treatment. In Fig 4F, for some volumes (>60 mL) our shear modulus fitting model did not converge and are excluded from the figure.

We explored the correlation between the intra-bladder pressure and UBV parameters as a function filling volume. Before formalin treatment, the coefficient of determination, *R*^2^, for the correlation between normalized pressure and normalized group velocity squared for the 3 pig bladders were *R*^2^ = 0.96, 0.94 and 0.97, respectively, and the *R*^2^ values between normalized pressure and normalized shear modulus were *R*^2^ = 0.97, 0.84 and 0.91. After formalin treatment, the correlation between normalized pressure and normalized group velocity squared were *R*^2^ = 0.99, 0.99 and 0.94 and; the corresponding values between normalized pressure and normalized shear modulus were *R*^2^ = 0.98, 0.97 and 0.97. The correlation coefficients demonstrate both the group velocity squared and shear modulus of the bladder correlate well with the intra-bladder pressure. The correlation coefficient for group velocity squared was higher than shear modulus.

### In Vivo UBV Measurements on Patients and Healthy Volunteers

**Fig 5:**
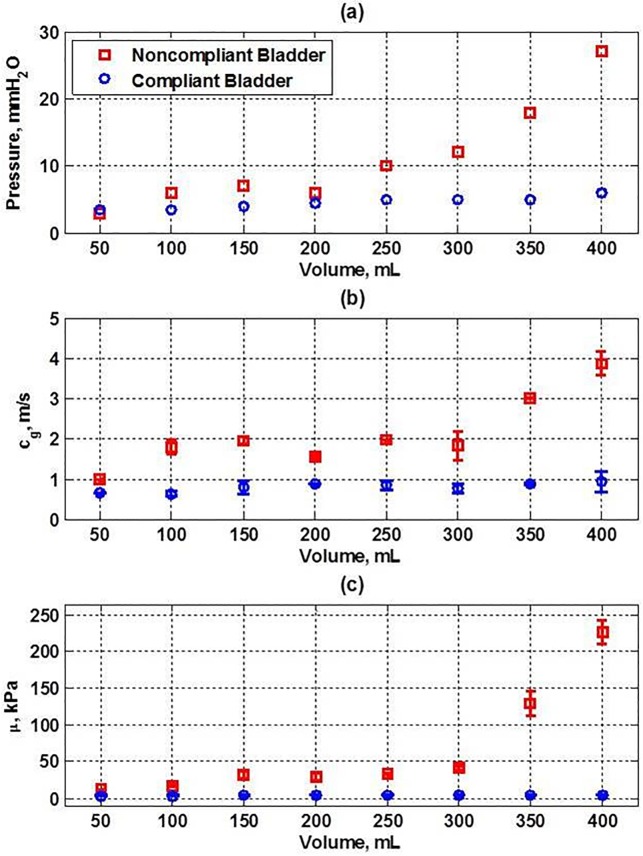
The summary of UDS and UBV measurements of two patients with a compliant (circles) bladder and another with a noncompliant (squares) bladder: (a) UDS measurements of the detrusor pressure versus filling volume, (b) UBV measurements of the group velocity (*c*_*g*_) versus volume, and (c) UBV measurements of shear modulus (*μ*) versus volume.

Concurrent in vivo UDS and UBV measurements of detrusor pressure, group velocity, and shear modulus as a function of filling volumes from our 2 patients are summarized in Fig 5. Fig 5 shows the mean value of 2 UDS measurements of detrusor pressure across the bladder wall as a function of the filling volume. The detrusor pressures in the noncompliant bladder are higher than in in comparison to the compliant bladder. Fig 5B shows the mean and standard deviation of the group velocity for Lamb waves travelling on opposite sides of the excitation ARF beam along the bladder wall as a function of volume is higher in the noncompliant than the compliant bladder. Similarly, Fig 5C shows the mean and standard deviation of the shear modulus for the noncompliant bladder is markedly higher than in the compliant bladder.

For the patient with noncompliant bladder, the correlation between normalized pressure and normalized square of the group velocity were *R*^2^ = 0.93; also, *R*^2^ = 0.99 between normalized pressure and normalized shear modulus. For the patient with compliant bladder, the correlation between normalized pressure and normalized square of the group velocity were *R*^2^ = 0.89; also *R*^2^ = 0.96 between normalized pressure and normalized shear modulus. These results suggest that both group velocity squared and shear modulus of the bladder wall can represent the same information as the detrusor pressure.

**Fig 6:**
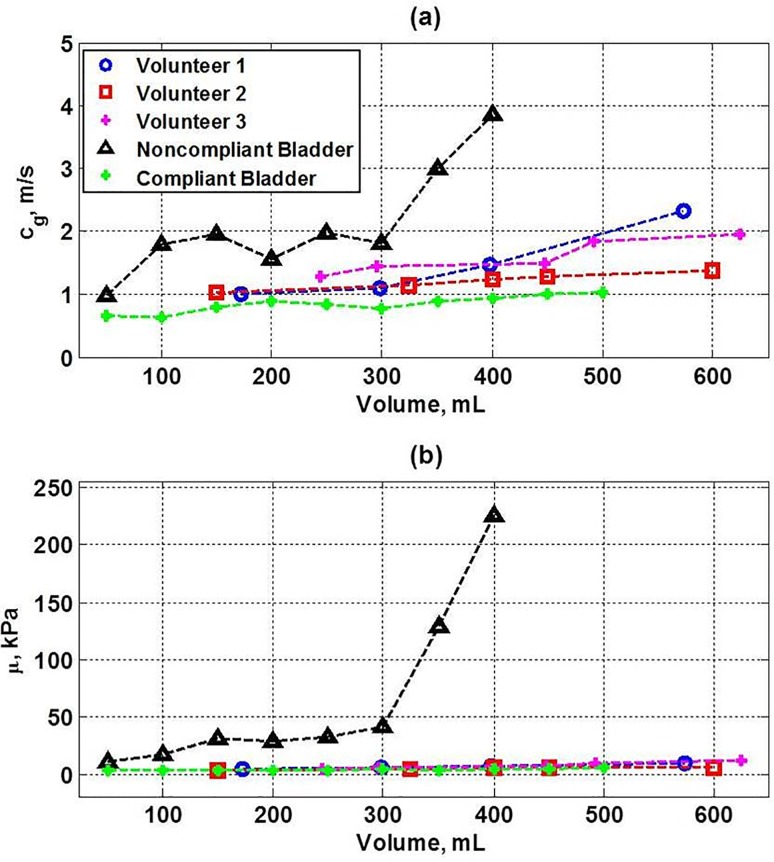
Plots of UBV parameters for the noncompliant bladder patient, compliant bladder patient and 3 healthy human volunteers: mean values of (a) group velocity values and (b) shear modulus as a function of filling volume.

The mean values of the UBV parameters as a function of filling volume for the 3 healthy volunteers and the two patients (Fig 5B and 5C) are plotted together in Fig 6. Fig 6 shows that the UBV parameters for the volunteers and the compliant bladder patient are relatively low and vary mildly with filling volume. In contrast, the UBV parameters of the noncompliant bladder patient are much higher and rise more rapidly in comparison to the compliant bladder patient and healthy volunteers.

## Discussion

We present an ultrasound-based method, ultrasound bladder vibrometry, for evaluation of the bladder wall mechanical properties as a function of filling volume.

The ex vivo experiments demonstrated the ability of the UBV to track changes of the bladder wall’s mechanical properties. For the porcine bladders, a high correlation (*R*^2^ > 0.83) between pressure measurements and UBV parameters suggest a strong linear relationship between shear modulus and the bladder intravesical pressure. This high correlation (*R*^2^ > 0.93) was maintained for the formalin treated bladder sample that was intended to mimic bladder stiffening.

High correlations were observed between UBV parameters and detrusor pressure measurements for 2 patients with different bladder compliance conditions (compliant and noncompliant) undergoing clinical UDS procedure. Both squared group velocity and shear modulus demonstrated high correlations (*R*^2^ > 0.88) with the detrusor pressure-volume curves, with shear modulus having a slightly higher correlation. The detrusor pressure increased mildly with filling volume for the compliant bladder patient in comparison to the noncompliant bladder patient. This is potentially due to the changes of the bladder wall thickness and its stiffness during filling. Increasing fluid in the bladder will decrease the thickness of the bladder wall as the tissue becomes taut, thus resisting further expansion that leads to increased intravesical pressure and in turn the detrusor pressure. While these pressure changes are mild in the compliant bladder patient, the noncompliant bladder cannot appropriately change volume to release the added pressure, which leads to a rapid increase in detrusor pressure. Similar observations can be seen in the shear modulus and group velocity curves for higher filling volumes in our noncompliant bladder patient. The high correlation between UBV parameters (squared group velocity and shear modulus) and detrusor pressure suggests that the UBV method can provide information on bladder compliance.

The application of UBV on healthy volunteers, using oral hydration and fractionated voiding, show similar group velocity and shear modulus characteristics in comparison to the compliant bladder patient. The UBV parameters of the healthy volunteers and the compliant bladder patient mildly increase in value with filling volume, whereas the UBV parameters of the noncompliant-bladder patient are more than 4 times greater at the end of filling. The similarity of the UBV parameters of the healthy volunteers to those of UDS-proven compliant bladder patient confirms the validity of UBV measurements obtained from the volunteers without catherization. Thus, our healthy volunteer experiments validate the capability of UBV as a truly noninvasive approach for evaluating bladder compliance.

### Advantages and Envisioned Clinical Utility of UBV

Currently, full UDS is the only means to assess bladder compliance. With further refinement and development of UBV, one could envision using this technology as an accurate, low cost, noninvasive tool for repetitive measurements, of this critical bladder functional parameter. The next iteration of UBV will automate this ultrasound technology to approximate the easiness and utility of the ubiquitous post-void bladder scan volume assessment. In this capacity, UBV will be capable of assessing bladder compliance not only in the setting of the chronic bladder neurogenic diseases such as pediatric myelomeningocele and spina bifida, but perhaps even in the setting of in-utero hydronephrosis assessment of unborn child’s bladder. In the pediatric population where full UDS is problematic and often futile, a quick, externally applied, unobtrusive device may offer tremendous advantage. In adults with evolving or resolving bladder dysfunction following spinal cord injury or chronic neurologic disease such as Parkinson’s disease or Multiple Sclerosis, UBV may be used as an index of progression or perhaps response to medical therapy. For more common voiding disorders (benign prostatic hyperplasia, underactive bladder, stress/urgency incontinence, and urinary retention) UBV may offer another parameter to help categorize these patients into responders or nonresponders to various medical/surgical management strategies. Urodynamics has not been used as a realistic serial exam in the short term due to its expense, invasiveness and relative complexity. To mimic the temporal catheter filling of the bladder used in UDS, one could copy the techniques used in normal volunteers arriving with a full bladder and voiding a defined volume with repeated UBV assessments. For those unable to void, the patient can arrive with full bladder and intermittently catheterize to prescribed volumes for serial UBV measures. The prospect of having a simple, accurate and reproducible UBV test could provide a new tool to apply a frequent measure of compliance in ways we never envisioned.

### Limitations

There are some limitations to our studies, which will require further investigation. First, the UDS detrusor pressure and volumes are measured continuously and any sudden changes that can be indication of a physiological condition such as detrusor overactivity are captured instantaneously. In contrast, the UBV measurements are acquired at discrete time points, thus it may miss intermittent detrusor contractions, detrusor overactivity. A possible solution is to acquire UBV data repeatedly at short intervals, e.g., every second, at each volume. Since UBV data acquisition is relatively fast, the system can be automated in the future to obtain sequential data acquisitions enabling the physician to measure transient events in the bladder wall. Second, the use of catheter in UDS allows the operator to repeat the test if necessary. Such freedom is not necessarily available to UBV. Although oral hydration is a convenient way to fill the bladder, it takes a relatively long time (approximately 2 hours) for bladder filling to be complete and stable (i.e., no additional urine will be accumulated during the test) for a repeat UBV data acquisition. Although this slow fill prevents the rapid fill artifact found with urodynamic studies, it limits repeated UBV administration. Third, elasticity is estimated via fitting a Lamb wave model and requires a precise estimation of the bladder wall displacement and thickness and might be a limiting factor if image quality is inadequate, specifically excessive abdominal fat could interfere with data acquisition. Currently, a software upgrade to enhance B-mode image quality of our ultrasound system is being developed to compensate for this technical problem.

Although our studies document that the UBV measurements accurately portray detrusor noncompliance [19,20], however it remains to be determined if UBV can detect findings such as detrusor overactivity (involuntary detrusor contractions during bladder filling) resulting in urge incontinence or vesicular/abdominal leak point pressure (the lowest pressures at which urinary leakage occurs) used in the clinical evaluation of stress urinary incontinence. There is no doubt that additional clinical studies on a larger group of patients are needed to overcome the aforementioned limitations and fully validate UBV as a clinical tool that could replace urodynamic testing for certain groups of patients.

## Conclusion

The feasibility of UBV as a noninvasive method to monitor the changes in the bladder wall mechanical properties is validated by the high correlation with pressure measurements in ex vivo porcine bladders and in vivo patient studies. Also, the similarities of the UBV parameters between healthy volunteers with normal bladder function and a patient with confirmed compliant bladder suggest that UBV can be conducted without catherization for a group of patients that can void voluntarily.

Comparison of the UBV results with the corresponding UDS results of patients with compliant and noncompliant bladders demonstrates the capability of UBV as a bladder compliance assessment tool. It is suggested that UBV may be used in place of UDS to monitor certain groups of patients at risk for developing noncompliant bladder. Further studies on a larger group of patients are needed to fully validate UBV as a clinical tool for assessment of bladder compliance.

## References

[1] Landau E, Jayanthi V, Churchill B, Shapiro E, Gilmour R, Khoury A, et al Loss of elasticity in dysfunctional bladders: urodynamic and histochemical correlation. The Journal of urology. 1994;152(2 Pt 2):702–5. 8021999 10.1016/s0022-5347(17)32685-x

[2] McGuire E, Woodside J, Borden T, Weiss R. Prognostic value of urodynamic testing in myelodysplastic patients. The Journal of urology. 1981;126(2):205–9. 7196460 10.1016/s0022-5347(17)54449-3

[3] Nitti V. Urodynamic and videourodynamic evaluation of the lower urinary tract In: Wein A KL, Novick A, Partin A, Peters C, editor. Campbell-Walsh urology. 10th ed. Philadelphia: Elsevier Saunders; 2011 pp. 1847–70.

[4] Wyndaele J, Gammie A, Bruschini H, De Wachter S, Fry C, Jabr R, et al Bladder compliance what does it represent: can we measure it, and is it clinically relevant? Neurourology and urodynamics. 2011;30(5):714–22. 10.1002/nau.21129 21661019

[5] Gormley EA, Lightner DJ, Burgio KL, Chai TC, Clemens JQ, Culkin DJ, et al Diagnosis and treatment of overactive bladder (non-neurogenic) in adults: AUA/SUFU guideline. The Journal of urology. 2012;188(6):2455–63. 10.1016/j.juro.2012.09.079 23098785

[6] Scarpero HM, Fiske J, Xue X, Nitti VW. American Urological Association Symptom Index for lower urinary tract symptoms in women: correlation with degree of bother and impact on quality of life. Urology. 2003;61(6):1118–22. 12809877 10.1016/s0090-4295(03)00037-2

[7] Scarpero HM, Padmanabhan P, Xue X, Nitti VW. Patient perception of videourodynamic testing: a questionnaire based study. The Journal of urology. 2005;173(2):555–9. 15643252 10.1097/01.ju.0000149968.60938.c0

[8] Denis M, Mehrmohammadi M, Song P, Meixner DD, Fazzio RT, Pruthi S, et al. Comb-Push Ultrasound Shear Elastography of Breast Masses: Initial Results Show Promise. 2015. 10.1371/journal.pone.0119398 PMC4361045 25774978

[9] Gennisson J-L, Deffieux T, Fink M, Tanter M. Ultrasound elastography: principles and techniques. Diagnostic and interventional imaging. 2013;94(5):487–95. 10.1016/j.diii.2013.01.022 23619292

[10] Mehrmohammadi M, Song P, Meixner DD, Fazzio RT, Chen S, Greenleaf J, et al Comb-push Ultrasound Shear Elastography (CUSE) for Evaluation of Thyroid Nodules: Preliminary In vivo Results. Medical Imaging, IEEE Transactions on. 2015;34(1):97–106. 10.1109/TMI.2014.2346498 PMC4280299 25122532

[11] Sarvazyan AP, Urban MW, Greenleaf JF. Acoustic waves in medical imaging and diagnostics. Ultrasound in medicine & biology. 2013;39(7):1133–46. 23643056 10.1016/j.ultrasmedbio.2013.02.006 PMC3682421

[12] Denis M, Bayat M, Mehrmohammadi M, Gregory A, Song P, Whaley DH, et al Update on breast cancer detection using comb-push ultrasound shear elastography. Ultrasonics, Ferroelectrics, and Frequency Control, IEEE Transactions on. 2015;62(9):1644–50. 10.1109/tuffc.2015.007043 PMC4687021 26688871

[13] Gregory A, Bayat M, Denis M, Mehrmohammadi M, Fatemi M, Alizad A, editors. An experimental phantom study on the effect of calcifications on ultrasound shear wave elastography. Engineering in Medicine and Biology Society (EMBC), 2015 37th Annual International Conference of the IEEE; 2015: IEEE. 10.1109/EMBC.2015.7319232 26737132

[14] Chen S, Urban MW, Pislaru C, Kinnick R, Zheng Y, Yao A, et al Shearwave dispersion ultrasound vibrometry (SDUV) for measuring tissue elasticity and viscosity. Ultrasonics, Ferroelectrics, and Frequency Control, IEEE Transactions on. 2009;56(1):55–62. 10.1109/TUFFC.2009.1005 PMC2658640 19213632

[15] Nenadic IZ, Qiang B, Urban MW, de Araujo Vasconcelo LH, Nabavizadeh A, Alizad A, et al Ultrasound bladder vibrometry method for measuring viscoelasticity of the bladder wall. Physics in medicine and biology. 2013;58(8):2675 10.1088/0031-9155/58/8/2675 23552842

[16] Nenadic IZ, Urban MW, Mitchell SA, Greenleaf JF. Lamb wave dispersion ultrasound vibrometry (LDUV) method for quantifying mechanical properties of viscoelastic solids. Physics in medicine and biology. 2011;56(7):2245 10.1088/0031-9155/56/7/021 21403186 PMC3086697

[17] Hasegawa H, Kanai H. Improving accuracy in estimation of artery-wall displacement by referring to center frequency of RF echo. Ultrasonics, Ferroelectrics, and Frequency Control, IEEE Transactions on. 2006;53(1):52–63. 10.1109/tuffc.2006.1588391 16471432

[18] Montaldo G, Tanter M, Bercoff J, Benech N, Fink M. Coherent plane-wave compounding for very high frame rate ultrasonography and transient elastography. Ultrasonics, Ferroelectrics, and Frequency Control, IEEE Transactions on. 2009;56(3):489–506. 10.1109/TUFFC.2009.1067 19411209

[19] Abrams P, Cardozo L, Fall M, Griffiths D, Rosier P, Ulmsten U, et al The standardisation of terminology of lower urinary tract function: report from the Standardisation Sub-committee of the International Continence Society. American journal of obstetrics and gynecology. 2002;187(1):116–26. 12114899 10.1067/mob.2002.125704

[20] O'Connell H, McGuire E. Leak point pressures. Urinary incontinence Urology, St Louis. 1997;93.

